# Unpacking the null: a post-hoc analysis of a cluster-randomised controlled trial of the WHO Safe Childbirth Checklist in Uttar Pradesh, India (BetterBirth)

**DOI:** 10.1016/S2214-109X(19)30261-X

**Published:** 2019-07-11

**Authors:** Megan Marx Delaney, Kate A Miller, Lauren Bobanski, Shambhavi Singh, Vishwajeet Kumar, Ami Karlage, Danielle E Tuller, Atul A Gawande, Katherine E A Semrau

**Affiliations:** a Ariadne Labs, Boston, MA, USA; b Brigham and Women's Hospital, Boston, MA, USA; c Harvard T H Chan School of Public Health, Boston, MA, USA; d Harvard Medical School, Boston, MA, USA; e Community Empowerment Lab, Lucknow, India

## Abstract

**Background:**

A coaching-based implementation of the WHO Safe Childbirth Checklist in Uttar Pradesh, India, improved adherence to evidence-based practices, but did not reduce perinatal mortality, maternal morbidity, or maternal mortality. We examined facility-level correlates of the outcomes, which varied widely across the 120 study facilities.

**Methods:**

We did a post-hoc analysis of the coaching-based implementation of the WHO Safe Childbirth Checklist in Uttar Pradesh. We used multivariable modelling to identify correlations between 30 facility-level characteristics and each health outcome (perinatal mortality, maternal morbidity, or maternal mortality). To identify contexts in which the intervention might have had an effect, we then ran the models on data restricted to the period of intensive coaching and among patients not referred out of the facilities.

**Findings:**

In the multivariable context, perinatal mortality was associated with only 3 of the 30 variables: female literacy at the district level, geographical location, and previous neonatal mortality. Maternal morbidity was only associated with geographical location. No facility-level predictors were associated with maternal mortality. Among facilities in the lowest tertile of birth volume (<95 births per month), our models estimated perinatal mortality was 17 (95% CI 11·7–24·8) per 1000 births in the intervention group versus 38 (31·6–44·8) per 1000 in the control group (p<0·0001).

**Interpretation:**

Mortality was not directly associated with measured facility-level indicators but was associated with general risk factors. The absence of correlation between expected predictors and patient outcomes and the association between improved outcomes and the intervention in smaller facilities suggest a need for additional measures of quality of care that take into account complexity.

**Funding:**

Bill & Melinda Gates Foundation.

## Introduction

Increasingly, we understand the need for improving the quality of care delivered by health-care systems worldwide. In 2016, of the 8·6 million people who died of conditions amenable to health care in lower-income and middle-income countries, 5 million (58%) presented for care but received poor-quality care.^1^ Simply increasing access to these health-care systems will not improve patient outcomes; the systems' ability to deliver high-quality care appropriately and consistently needs to be improved.

To combat maternal and perinatal mortality that remain untenably high, WHO developed the Safe Childbirth Checklist. Designed for use during childbirth, the Checklist reminds birth attendants of 28 evidence-based practices that can prevent complications, identify emergencies early, and ensure a base of high-quality care.^2^ Studies have demonstrated that using the Checklist can increase birth attendants' adherence to evidence-based practices,^3^ but until 2017, there was no evidence about the effect of the Checklist on maternal and neonatal outcomes.

The BetterBirth trial,^4^ a large-scale, matched-pair, cluster-randomised controlled trial, tested a coaching-based implementation of the Checklist in primary and community health centres in Uttar Pradesh, India. Measured outcomes included adherence to evidence-based practices and health outcomes (maternal mortality, perinatal mortality, and severe maternal morbidity within 7 days of birth). Although the intervention resulted in increased performance of evidence-based practices, it did not affect any maternal or perinatal health outcome.^5^

**Figure 1:**
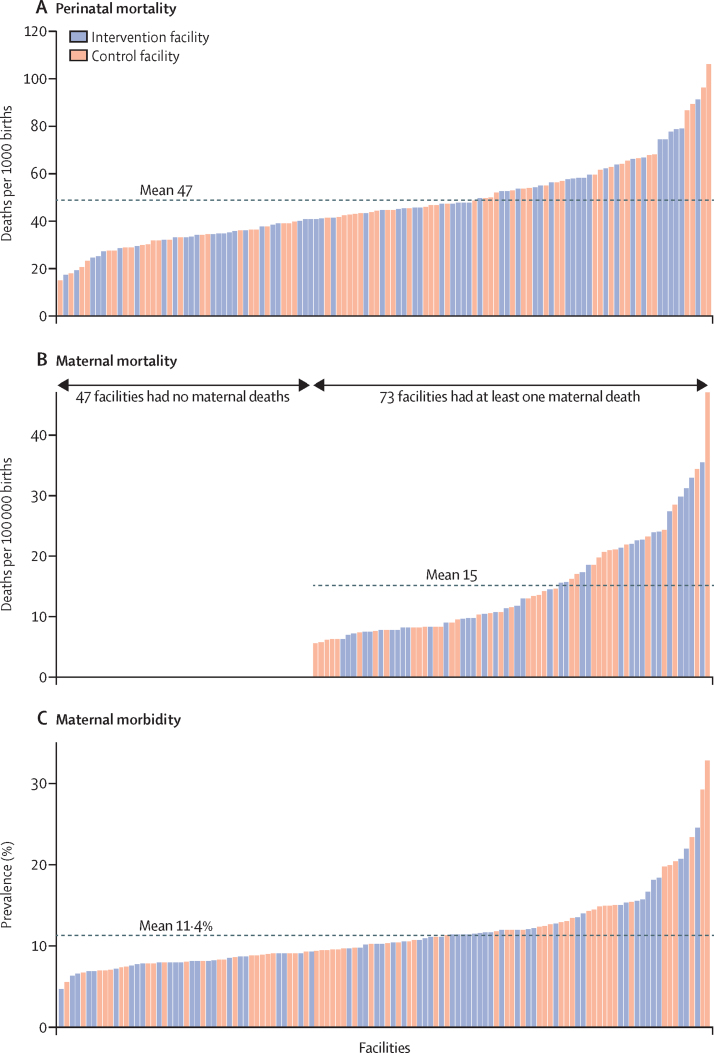
Variation in perinatal mortality (A), maternal mortality (B), and prevalence of maternal morbidity (C) at the facility level Each column represents one of the 120 facilities included in the BetterBirth trial, sorted by outcome.

Why didn't the intervention affect maternal and neonatal outcomes? Other research^2^ has shown the ability of the individual Checklist practices to improve specific health outcomes. In the BetterBirth trial, by contrast, no individual practice strongly correlated with patient outcomes, but total count of practices implemented during a birth did.^6^ Moreover, dramatic variation of health outcomes across the 120 facilities in the study (figure 1) also suggested meaningful differences in quality of care between facilities^5^ that go beyond measurable differences in practices. Possibly, in facilities where more practices are performed, care is better overall, and outcomes reflect that higher quality, regardless of which evidence-based practices were used.^6^

**Research in context**

**Evidence before this study**

In 2017, the *Lancet Global Health* Commission on High-Quality Health Systems highlighted the enormous human and financial burden of poor-quality care and called for improved measurement to increase accountability and meaningfully track progress. Easily measured inputs such as supply and staff availability have not been shown to be closely correlated with quality of care delivered, yet process measures, such as direct observation of care, can increase the reporting burden for health-care systems that might already have too few clinicians. We searched MEDLINE with the terms “quality (maternal OR obstetric OR delivery OR intrapartum) care”, “measurement”, “India OR developing countries”, and “mortality” for articles published in English between Jan 1, 2008, and Jan 1, 2018. Despite persistent calls for improved measure of quality of care in this literature, no consensus has been reached on the most useful indicators. In addition, quality-of-care interventions focused on improving adherence to evidence-based practices at the point of care often fail to save lives at scale.

**Added value of this study**

The BetterBirth trial was a cluster-randomised controlled trial that tested the effect of a coaching-based implementation of the WHO Safe Childbirth Checklist in Uttar Pradesh, India. It found that the intervention increased birth attendants' adherence to evidence-based practices during childbirth, yet had no effect on mortality. The wide variation in mortality at similarly resourced, public primary health centres in Uttar Pradesh prompted us to further investigate potential facility-level characteristics that correlate with mortality. In this post-hoc analysis of the BetterBirth trial data, we analysed 30 variables collected during the trial that could potentially explain the variation in mortality. This analysis is in response to a paucity of published literature on the relationship between the quality of childbirth care and mortality in settings with high maternal and perinatal mortality, such as Uttar Pradesh.

**Implications of all the available evidence**

As rates of facility-based childbirth continue to increase globally, it is essential that policy makers, implementers, and researchers remain aware that improvements in easy-to-assess input and process measures might not produce the hoped-for reductions in childbirth-related mortality. Quality-related indicators measured in this large-scale randomised controlled trial were not correlated with mortality. We must consider the need to measure, intervene on, or differentially target interventions based on factors that, thus far, have not consistently been considered or measured.

To improve the characterisation of the various contexts in the BetterBirth trial, we posed two post-hoc research questions. First, what characteristics correlated with facility-level outcomes? Regardless of the potential success of the BetterBirth intervention, facility characteristics that are systematically related to outcomes could suggest causal pathways or hypotheses for future work. Second, although the BetterBirth intervention did not have an overall effect on maternal and neonatal outcomes, did it improve outcomes at certain sites, under intensive coaching, or under any other identifiable circumstances? We did a subgroup analysis, focused on the period of intensive coaching and on the patients treated in facility, excluding referrals. In this subgroup, we assessed differential responses to the intervention based on facility characteristics.

## Methods

### Study design

In this study we did a post-hoc analysis of the results of the BetterBirth trial, which is registered at ClinicalTrials.gov, number NCT02148952. The BetterBirth trial^4^, ^5^, ^7^, ^8^, ^9^ was a matched-pair, cluster-randomised controlled trial testing the effect of a coaching-based WHO Safe Childbirth Checklist programme on a composite outcome of perinatal death, maternal death, and maternal severe morbidity in primary and community health centres in Uttar Pradesh, India. The 8-month intervention involved 43 day-long coaching visits to support Checklist use and to problem solve around barriers to quality maternal and neonatal care. Supplies and clinical training were not provided. Coaching intensity decreased over time from twice per week in the intensive phase (first 4 months) to weekly to monthly in the taper phase (second 4 months). We initiated outcome data collection after the completion of 2 months of coaching and continued outcome data collection for 4 months after the end of the intervention (total of 12 months). The post-coaching portion of the data collection period (4 months) involved no coaching visits to assess intervention sustainability. The BetterBirth intervention,^7^, ^8^ study protocol,^4^ data quality,^9^ and results^5^ have been described in detail elsewhere.

For administrative purposes of the study, we delineated five geographically defined regional hubs throughout Uttar Pradesh: central, southwest, southeast, northeast, and west. Each hub administered the BetterBirth trial in approximately twenty facilities across three to six districts. These hubs were created out of convenience and included both rural and urban sites. We included the term hub in our statistical model to understand potential differences in how the programme was deployed across the state.

Through telephone and in-person follow-up, we collected 7-day outcomes for 157 145 mother–infant pairs from 60 intervention and 60 control facilities. We measured mortality through both facility records and patient (or surrogate) self-report, and we measured maternal severe morbidity through patient (or surrogate) self-report. Facility staff could refer both mothers and infants out of the study facility, after which their care was neither recorded nor affected by the study protocol; all referred cases were followed for health outcomes.

We sought and received consent from state-level and facility-level leadership to collect deidentified data on all eligible women in facility registers. Birth attendants obtained verbal consent for 7-day follow-up from labouring women or their surrogates at the time of facility discharge, and data collectors reconfirmed consent at follow-up. The study protocol was approved by ethics review boards at Community Empowerment Lab, Jawaharlal Nehru Medical College, the Harvard TH Chan School of Public Health, Population Services International, WHO, and the Indian Council of Medical Research.

### Procedures

In this post-hoc analysis, we calculated facility-level perinatal mortality as the number of perinatal deaths (infant death within 7 days of birth, including stillbirths) per 1000 births over a given period. We calculated maternal severe morbidity as the number of women experiencing seizures, loss of consciousness for more than 1 h, fever with foul-smelling vaginal discharge, haemorrhage, or stroke within 7 days of birth per 100 births (%) over a given period. Maternal deaths (within 7 days of birth) were extremely rare; many facilities experienced no maternal deaths at all. Therefore, we measured maternal mortality as a binary outcome, indicating whether the facility experienced any maternal deaths over the course of the study.

Since this is a post-hoc analysis, study data was not designed to answer our specific research questions. Rather, we drew from the BetterBirth data all possible measures of the four levels of the childbirth ecosystem: the woman and her community; the birth attendant and their perspectives, training, and experience; the facility and its resources, staff, leadership, and culture; and the health system and its resources and connections. Under this framework, we identified and included a set of 30 characteristics that were measured in both intervention and control facilities (table 1).

**Table 1:** Definitions of all facility measures and distributions by group

		**Intervention facilities (n=60)**	**Control facilities (n=60)**
**Women and community measures**			
Female literacy in district*		58·6% (5·3)	59·4% (5·3)
Higher-income districts†		17 (28%)	18 (30%)
Geographical location in central study hub‡		19 (32%)	19 (32%)
Proportion of patients aged ≥35 years§¶		2·1% (1·9)	2·1% (1·7)
Proportion of patients age ≤25 years§¶		37·8% (8·1)	38·3% (8·9)
Proportion of patients in scheduled caste¶		29·4% (8·9)	32·4% (8·5)
Proportion of patients in Other Backward Caste¶		47·4% (11·7)	46·5% (9·0)
Mean patient gravida¶‖		2·4 (0·2)	2·4 (0·2)
Proportion of patients with any of 14 complications**¶‖		3·4% (3·4)	2·5% (2·3)
Proportion of patients with anaemia or haemoglobin issues¶‖		2·6% (6·8)	2·0% (6·4)
**Birth-attendant measures**			
Age of birth attendants, years		37·4 (5·0)	36·9 (5·3)
Number of years since last training‖		4·7 (2·8)	4·5 (1·9)
Number of years of experience‖		9·8 (4·6)	10·2 (5·0)
Proportion of birth attendants trained††			
	<50% (reference category)	35 (58%)	31 (52%)
	≥50%	16 (27%)	23 (38%)
	100%	9 (15%)	6 (10%)
Any birth attendants in scheduled caste‡‡		28 (47%)	41 (68%)
Proportion of birth attendants in Other Backward Caste		41·6% (22·1)	45·8% (24·1)
**Primary-level facility measures**			
Facility type			
	Primary health centre (reference category)§§	23 (38%)	23 (38%)
	Community health centre	27 (45%)	29 (48%)
	Community health centre first-referral unit	10 (17%)	8 (13%)
Previous neonatal mortality¶¶		2·1% (1·0)	1·7% (0·8)
Total number of staff‖‖		4·4 (1·2)	4·4 (1·1)
Types of staff at facility‖‖			
	Nurses only (reference category)	22 (37%)	22 (37%)
	Nurses and auxiliary nurse midwifes	22 (37%)	19 (32%)
	Nurses and female medical officers	10 (17%)	8 (13%)
	Other staff pattern	6 (10%)	11 (18%)
Birth volume (number of births during study period)¶***		1330 (412)	1289 (345)
Time between admission and delivery, min¶		204 (59)	204 (50)
Proportion of caesarean-section deliveries¶‖		0·2% (0·6)	0·1% (0·5)
≥50% of deliveries attended by one nurse alone¶†††‡‡‡		40 (67%)	40 (67%)
≥50% of deliveries attended by one auxiliary nurse midwife alone¶‡‡‡§§§		5 (8%)	4 (7%)
Any deliveries ever attended by a doctor, whether with other clinicians or alone¶§§§		33 (55%)	39 (65%)
Number of supplies available (of 28¶¶¶)		21·9 (1·9)	20·9 (1·8)
Number of essential medicines available (of 4‖‖‖)		2·8 (0·7)	2·6 (0·7)
**Systemness measures**			
Proportion of referrals¶		7·3% (6·2)	6·4% (5·7)
Distance to district hospital, km		30 (14)	30 (12)

Data are n (%) or mean (SD), where n is the number of sites.

* Data on the proportion of district literacy taken from government statistics in Uttar Pradesh (2014).

† Defined as average annual income greater than US$720; the continuous variable of mean income in district was converted to binary because the distribution is not normal, with distinct high and low clusters, and the cutoff point of $720 is the midpoint between the clusters. Data on annual income taken from government statistics in Uttar Pradesh (2014).

‡ The central region centred on Lucknow and had different outcomes compared with the four other hubs, which were more similar to each other.

§ Age categories are used instead of overall mean patient age because they are more strongly correlated with outcomes than the mean age at a facility.

¶ Value is aggregated from patient level, so it changes with different time spans.

‖ Distribution is skewed so in statistical models and tests, variable is logged to approach normality.

** The complications are: antepartum haemorrhage, eclampsia, leaking, breech presentation, no labour pain, weak uterus, membrane absent, low baby weight, previous caesarean section, previous low-segment caesarean section, placenta previa, hypertension, hydrocephaly, and cephalopelvic disproportion.

†† Continuous variable was converted to categorical because of non-normal distribution (had clusters at 0% and 100%).

‡‡ Continuous variable was converted to binary because of non-normal distribution (had large cluster at 0% and a few facilities at more than 0%).

§§ Primary health centre category includes block-level primary health centre and primary health centre.

¶¶ The number of babies who died in the facility (before discharge or referral) divided by the total number of births during the 12 months preceding the start of study; this value, which was captured retrospectively from facility birth registers, is not directly comparable with the BetterBirth perinatal mortality, which tracks outcomes past discharge up to day 7, includes deaths that occurred outside the study facility, and is based on data collected under a stringent quality assurance protocol.

‖‖ Measured at start of study only.

*** Count of births during study period, from study data.

††† Continuous variable was converted to binary because of non-normal distribution (had a large cluster above 80% and a smaller one at 0%).

‡‡‡ 0% does not mean that no staff attended the delivery, but that the delivery was not attended by one nurse or auxiliary nurse midwife working alone; the birth could have been attended by more than one staff or a different combination of staff.

§§§ Continuous variable was converted to binary because of non-normal distribution (had a large cluster at 0%).

¶¶¶ The 28 supplies were: antibiotics for the baby, antibiotics for the mother, baby scale, baby warmer, bag and mask, BCG vaccine, blood pressure cuff, clean blade or scissors, clean gloves, clean pads, clean towel, cord tie or clamp, fetoscope or doppler, HIV testing kit, intravenous fluid bag, magnesium sulphate, mucus extractor or aspirator, nevirapine for the baby, nevirapine for the mother, oxytocin, partograph, polio vaccine, soap and water or alcohol rub, sterile needles and syringes, stethoscope, thermometer, urine dip sticks, and vitamin K.

‖‖‖ The 4 medicines were: vitamin K, magnesium sulphate, oxytocin, and antibiotics (for mother or baby).

Several of these characteristics, such as facility type or staffing pattern, were assessed at the beginning of the intervention period and were treated as static. Others were patient-level or delivery-level characteristics that we drew from our study data and aggregated to the facility level. For example, we calculated “proportion of patients in scheduled caste” as the percentage of patients over a given period at the facility who were in that caste, a value that could change depending on the period selected (for example, the entire study period *vs* the intensive-coaching phase).

We calculated the value of each facility measure by study group. For percentages and continuous variables, we report means and standard deviations across all facilities in each group. For counts of facilities, we report the number and percentage of facilities in each group. Because of the risk of type I error inflation, we did not calculate p values for differences across groups in these comparisons.

### Statistical analysis

For this post-hoc analysis, we constructed multivariable models for each of the three outcomes and for both research questions. First, we calculated bivariate associations between the outcome and all 30 covariates, adjusted for matching, using Pearson's correlation for continuous variables and Rao-Scott χ^2^ test for categorical variables. All covariates showing significant (p<0·05) bivariate relationships with the outcome were selected for inclusion in the multivariable models. This 0·05 threshold (rather than 0·10) represented a balance between including important predictors in the models and conserving the statistical power in our sample of 120 facilities.

To test correlations between facility-level measures and outcomes, the models used data from all births across the entire study period, aggregated to the study-site level. We used ordinary least squares regression models for the outcomes of perinatal mortality and percentage of women with severe maternal complications. We used logistic regression to model the binary outcome of any maternal death.

To identify subgroups in which the intervention might have had an effect, the models used data from the intensive-coaching phase only (intensive coaching occurred for 4 months; however, outcome data was only collected after the first 8 weeks of coaching occurred). Also, we excluded cases that were referred out to a higher level of care because the BetterBirth study had no presence in the referral facilities; although morbidity was higher in the referred population than in the rest of the population, any effect of the intervention itself would be indirect and attenuated. We did not fit a model for maternal mortality because only three facilities had any maternal deaths once referred cases were excluded. We checked each of the previously selected predictors for interactions with study group by modelling:

outcome=group+predictor+(group×predictor).

We included in the final multivariable models any interaction terms with p<0·05.

To address the risk of false discovery (inflated type I error), we first set α at 0·05 for each research question, then applied a Bonferroni adjustment across the family of models that address that question. We considered parameter estimates significant if

$$p<\frac{0.05}{k_{perinatalmortality}{+}_{maternalmorbidity}+k_{anymaternalmortality}},$$

where *k*_outcome_ is the number of covariates selected for the multivariable model for that outcome.

Some predictors required transformation to better suit the assumptions of multivariable models. Six continuous measures (mean patient gravida, percentage of patients with any of 14 complications, percentage of patients with anaemia or haemoglobin issues, mean years since last training, mean years of experience, and percentage of caesarean-section deliveries) had skewed distributions, so we took their natural logs to bring them closer to normality. For ease of interpretation, we present the unlogged versions of these six measures in table 1. Six continuous measures (higher income district; proportion of birth attendants trained; any birth attendants in scheduled caste; half or more of deliveries attended by one nurse alone; half or more of deliveries attended by one auxiliary nurse midwife alone; and any deliveries ever attended by a doctor, whether with other clinicians or alone) had highly non-normal distributions, so we converted them to binary or ordinal variables, as defined in table 1. We also tested the residuals of all ordinary least squares models for normality and homoskedasticity. Although the residuals were normally distributed, they displayed borderline heteroskedasticity in three models. To adjust for this, we took the natural log of the dependent variables, as noted in the tables of results.

### Role of the funding source

The funder of the study reviewed the study design and sample size calculations, but played no role in data collection, data analysis, data interpretation, or writing of the report. All authors had full access to all the data in the study and MMD, KAM, and KEAS had final responsibility for the decision to submit for publication.

## Results

Nearly all facility-level measures were balanced between the study groups (intervention and control sites), reflecting the matched-pair design and the facility-level randomisation (table 1). The few with differences, such as “Any birth attendants in scheduled caste” or “any deliveries ever attended by a doctor”, were probably the result of chance.

The set of three models investigating correlations between facility-level measures and outcomes contained a total of 32 predictors (table 2), so the adjusted α was 0·05/32=0·0016. By this threshold, only three predictors correlated significantly with perinatal mortality in the multivariable context: proportion of female literacy in the district (inverse association), location in the central hub, and neonatal mortality in the 12 months before the study. One predictor correlated significantly with severe maternal morbidity: location in the central hub. No predictors correlated significantly with maternal mortality.

**Table 2:** Multivariable model results, including referred cases and all study phases

		Perinatal mortality*(n=95†**)**		Severe maternal morbidity*‡**(n=100**†**)**		**Any maternal deaths**§**(n=114**†**)**	
		Mean change (95% CI)	p value	Mean change (95% CI)	p value	Odds ratio (95% CI)	p value
**Women and community measures**							
Proportion of female literacy in district		−1·53 (−2·37 to −0·70)	0·00064	−0·79 (−1·87 to 0·28)	0·14	0·9 (0·8 to 1·0)	0·17
Higher-income district		0·01 (−0·13 to 0·14)	0·91	−0·04 (−0·14 to 0·06)	0·45	..	..
Geographical location in central study hub		0·26 (0·13 to 0·40)	0·00028	0·33 (0·19 to 0·47)	<0·0001	..	..
Proportion of patients aged ≥35 years		0·39 (−3·38 to 4·16)	0·84	0·52 (−2·71 to 3·74)	0·75	..	..
Proportion of patients aged ≤25 years		..	..	−0·04 (−0·77 to 0·69)	0·92	..	..
Proportion of patients in Other Backward Caste		..	..	−0·16 (−0·71 to 0·39)	0·56	..	..
Mean patient gravida		..	..	0·87 (0·22 to 1·51)	0·0094	11·2 (1·3 to 98·7)	0·030
Proportion of patients with any of 14 complications		..	..	..	..	1·1 (0·9 to 1·4)	0·42
Proportion of patients with anaemia or haemoglobin issues		..	..	..	..	1·0 (0·9 to 1·1)	0·86
**Birth-attendant measures**							
Mean age of birth attendants		−0·00 (−0·02 to 0·01)	0·63	−0·02 (−0·03 to −0·01)	0·0072	..	..
Number of years since last training		0·10 (0·01 to 0·19)	0·031	..	..	..	..
Number of years of experience		0·03 (−0·15 to 0·21)	0·73	0·16 (0·02 to 0·30)	0·025	..	..
Proportion of birth attendants trained (reference <50%)		..	..	..	0·039	..	..
	≥50%	..	..	−0·16 (−0·30 to −0·02)	..	..	..
	100%	..	..	−0·19 (−0·36 to −0·02)	..	..	..
Proportion of birth attendants in Other Backward Caste		..	..	0·02 (−0·27 to 0·30)	0·91	..	..
**Primary-level facility measures**							
Previous neonatal mortality		10·64 (4·82 to 16·46)	0·00065	3·64 (−2·77 to 10·05)	0·26	..	..
Birth volume (number of births during study period)		..	..	0·00 (0·00 to 0·00)	0·17	1·001 (1·0001 to 1·003)	0·033
Proportion of caesarean-section deliveries		−0·06 (−0·11 to −0·02)	0·0070	..	..	0·6 (0·3 to 1·3)	0·20
≥50% of deliveries attended by one nurse alone				0·03 (−0·08 to 0·14)	0·58	..	..
≥50% of deliveries attended by one auxiliary nurse midwife alone		−0·14 (−0·37 to 0·09)	0·21	..	..	..	..
Number of essential medicines available (of 4)		..	..	0·07 (0·02 to 0·13)	0·014	..	..
**Systemness measures**							
Proportion of referrals		..	..	..	..	1·2 (1·0 to 1·4)	0·020

Details on each measure are described in table 1. In this set of models, *k*_perinatal mortality_=10, *k*_maternal morbidity_=15, and *k*_any maternal mortality_=7, so the adjusted p value threshold for the statistical tests in this table is 0·05/32=0·0016.

* Dependent variable was logged to ensure normality and homoskedasticity of residuals; for this outcome, R^2^=0·43 and the number of predictors was 10.

† n is below 120 because of missing data for some covariates.

‡ Dependent variable was logged to ensure normality and homoskedasticity of residuals; for this outcome, R^2^=0·58 and the number of predictors was 15.

§ For this outcome, Akaike information criterion was 0·58 and the number of predictors was 7.

The set of two models investigating where the intervention might have had an effect contained a total of 21 predictors (table 3), so the adjusted α is 0·05/21=0·0024. We included two interaction terms with group in the final model for perinatal mortality: location in the central hub and birth volume. We included two interaction terms with group in the final model for maternal severe morbidity: availability of four essential medicines and percentage of patients aged 35 years or older. With perinatal mortality as an outcome, location in the central hub was again a significant correlate (p=0·0017), but the interaction term lost significance in the multivariable context (p=0·10). Given the reduced α threshold, these results do not support a conclusion that the intervention worked differently in the central hub than in other study hubs, once all else was controlled for. The only other significant predictor was for percentage of caesarean-section deliveries, but there was no indication of an interaction with study group.

**Table 3:** Multivariable model results, including only the intensive-coaching phase and excluding referrals, with significant interaction terms with study group

		Perinatal mortality*(n=105†**)**		**Severe maternal morbidity**†**(n=104**‡**)**	
		Mean change (95% CI)	p value	Mean change (95% CI)	p value
**Study arm**					
Intervention group		−1·14 (−1·73 to −0·55)	0·00033	0·05 (−0·01 to 0·11)	0·089
**Women and community measures**					
Proportion of female literacy in district		−1·97 (−4·57 to 0·62)	0·13	−0·16 (−0·29 to −0·02)	0·021
Higher-income district		..	..	−0·00 (−0·02 to 0·02)	0·99
Geographical location in central study hub		0·34 (0·13 to 0·54)	0·0017	0·04 (0·02 to 0·05)	<0·0001
Interaction with group: intervention sites located in central study hub		−0·37 (−0·82 to 0·08)	0·10	..	..
Proportion of patients aged ≥35 years		..	..	0·30 (−0·29 to 0·88)	0·32
Interaction with group: proportion of patients aged ≥35 years at intervention sites		..	..	−0·75 (−1·54 to 0·04)	0·061
Proportion of patients aged ≤25 years		..	..	−0·06 (−0·16 to 0·03)	0·20
Mean patient gravida		..	..	0·06 (−0·01 to 0·13)	0·11
**Birth-attendant measures**					
Proportion of birth attendants trained (reference <50%)		..	..	..	0·011
	≥50%	..	..	−0·02 (−0·04 to −0·01)	..
	100%	..	..	−0·02 (−0·04 to −0·00)	..
**Primary-level facility measures**					
Previous neonatal mortality		13·44 (3·55 to 23·33)	0·0088	..	..
Birth volume (number of births during study period)		−0·03 (−0·16 to 0·10)	0·61	..	..
Interaction with group: birth volume at intervention sites		0·42 (0·17 to 0·66)	0·0012	..	..
Proportion of caesarean-section deliveries		−0·13 (−0·22 to −0·05)	0·0017	..	..
≥50% of deliveries attended by one nurse alone		..	..	0·02 (0·01 to 0·04)	0·0013
Number of essential medicines available (of 4)		..	..	0·03 (0·01 to 0·04)	0·00043
Interaction with group: number of essential medicines available (of 4) at intervention sites		..	..	−0·02 (−0·04 to 0·00)	0·080
**Systemness measures**					
Proportion of referrals		−2·06 (−4·21 to 0·09)	0·060	..	..

Details on each measure are described in table 1. In this set of models, *k*_perinatal mortality_=9 and *k*_maternal morbidity_=12, so the adjusted p value threshold for these statistical tests is 0·05/21=0·0024.

* Dependent variable was logged to ensure normality and homoskedasticity of residuals; for this outcome, R^2^=0·43 and the number of predictors was 9.

† For this outcome, R^2^=0·55 and the number of predictors was 12.

‡ n is below 120 because of missing data for some covariates.

The base term for study group was significant and negative (mean change −1·14, 95% CI −1·73 to −0·55; p=0·00033), suggesting decreased perinatal mortality in intervention sites, when we controlled for all other factors. Yet the interaction term for group and birth volume was significant (p=0·0012) at the adjusted α level (α=0·0024). The parameter was positive (0·42, 0·17 to 0·66), suggesting increased perinatal mortality at facilities with increased volumes, but only among sites in the intervention group. The base term for birth volume was not significant, suggesting there was no relationship between birth volume and outcomes, independent of study group.

**Figure 2:**
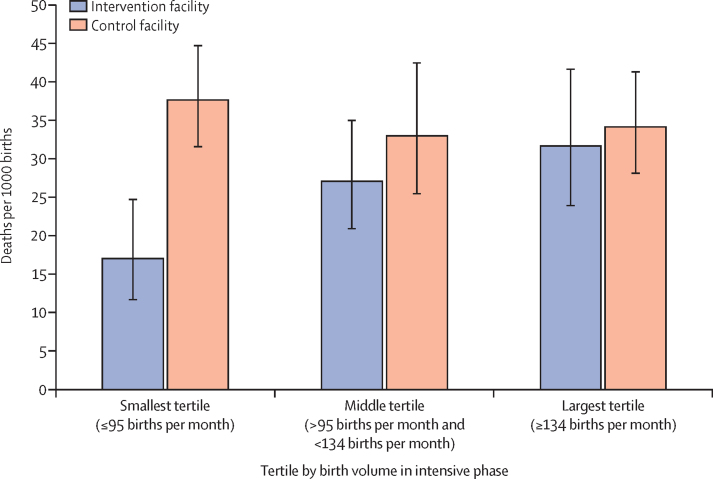
Estimated perinatal mortality by group and tertile of birth volume Mean (95% CI) estimates were based on multivariable models, adjusted for the proportion of female literacy in the district, geographical location in central region, previous neonatal mortality, proportion of referrals, and proportion of caesarean-section deliveries; the analysis was restricted to the intensive-coaching phase, excluding referrals. Each tertile includes 40 facilities.

To aid interpretation of this interaction, we converted the birth-volume variable to tertiles of 40 facilities each, with the lowest tertile having 95 monthly births or fewer in the intensive study phase and the highest tertile having 134 or more monthly births in the intensive study phase. With this form of the birth-volume variable, the perinatal-mortality model (parameters not shown) generated the same substantive results as the models presented earlier in this Article (table 3). Modelled estimates of perinatal mortality by group and tertile of birth volume at the mean values of all other predictors suggest that the intervention might have reduced perinatal mortality at the sites in the lowest birth-volume tertile (figure 2). This result disappeared during the taper and post-coaching phases (data not shown). Modelling these estimates with birth volume per staff member resulted in an interaction term that was similar in direction but smaller in magnitude and somewhat less significant than with total birth volume per site (data not shown).

The model of severe maternal morbidity found no significant interactions with study group (table 3). Base terms for location in the central hub, essential medicine availability, and “≥50% of deliveries attended by one nurse alone” reached significance.

## Discussion

In the BetterBirth trial,^5^ we found that, despite birth attendants' increased adherence to evidence-based practices, health outcomes remained unaffected across over 157 000 mother–neonate pairs. However, we found up to a seven-time variation in mortality and prevalence of morbidity across the 120 public primary and community health centres in the trial. In this post-hoc analysis of facility-level outcomes, we found that mortality and prevalence of morbidity did not correlate with expected factors such as staff experience or supply availability but did with distal risk factors such as low proportions of female literacy and geographical location.

Perhaps our most useful results, however, were the more proximal factors that did not correlate with outcomes, such as staffing patterns, provider education, patient-population characteristics, proportion of caesarean-section deliveries, and type of facility. The assumption that care can be measured and improved via these factors is proving to be oversimplified, not only in maternal and child health but also in global health work more generally. Birth attendants require adequate knowledge and skill to perform evidence-based practices, but they also need motivation and empowerment to act in the best interests of their patients, both in normal care and in crises. When social hierarchies or informal incentive systems are in play, birth attendants might not be able to act quickly and decisively to prevent poor outcomes. By contrast, work in surgery^10^ has demonstrated that quality-culture factors such as respect for front-line staff and leadership quality correlate with reduced mortality. Similarly, facilities must have supplies and trained staff, but they might also require the more difficult to measure qualities of physical and social safety^11^, ^12^ and well functioning teams.^13^ Finally, the cohesion and seamless functioning of the health system is more than just the sum of its parts: for example, both primary and secondary facilities might independently provide high-quality care, but without shared communication and accountability^14^ (both unmeasured), patients referred from one facility to another continue to be at risk of poor outcomes.

Improving these conventionally measured factors and interventions to change point-of-care behaviour are also likely to be insufficient to improve health outcomes. At minimum, once we learn to measure new factors such as motivation, leadership, and shared accountability, we need to ensure that those aspects of health systems are functioning effectively and efficiently. We might need to supplement more traditional quality-of-care interventions with development efforts in education and poverty reduction to have a large-scale impact on health outcomes.

Even so, we believe a role remains for interventions seeking to directly improve the delivery of care, in addition to large, systems-level changes. We found that the BetterBirth programme decreased perinatal mortality at lower volume facilities during the most intensive coaching period (with referred cases excluded, controlling for other factors). Several hypotheses could explain this finding. Birth volume might not be a meaningful factor itself but might be confounded with some other facility characteristic driving mortality variation. Alternatively, at facilities with low patient volumes, birth attendants might have more time and cognitive space to accommodate new evidence-based practices. In high-volume sites, birth attendants might be too busy to implement the Checklist, especially if some or all of its potentially overwhelming 28 practices are unfamiliar. Research on crowding in labour floors and emergency rooms in high-income countries is consistent; facilities have worse outcomes during high-volume periods.^15^, ^16^ Another possible explanation is that coaches might have been able to create more effective culture change at facilities with fewer staff members. Fewer staff could mean fewer detractors to overcome or simply smaller required effort and coordination, so coaches might have found it easier to build support for Checklist use at smaller facilities.

If quality-of-care interventions such as the BetterBirth programme can be more effectively implemented at lower volume facilities, such facilities might be the natural targets of similar quality-of-care interventions. Regardless of mechanism, when seeking to introduce the Checklist (or any quality-of-care intervention) at a facility, assessing the facility's readiness to adopt an intervention—including the cognitive load on the birth attendants and the staff's openness to change—can provide guidance in adapting the implementation package. For instance, in a busy facility, it might be more effective to introduce evidence-based practices sequentially rather than all at once. In research contexts, this result suggests a use for as-treated analyses: in the initial study design for the BetterBirth trial, we attributed health outcomes of referred-out cases to the initial study facility. However, we had no information on the quality of care at the secondary and tertiary facilities nor on the method of transit. The lack of details limited our interpretation of these cases.

Limitations to this study include dataset size and measurement constraints. Although the patient-level dataset for the BetterBirth trial was large, the facility-level dataset is relatively small (120 facilities), which limited our statistical power. Additionally, we could not link the performance (or non-performance) of evidence-based practices to health outcomes at the facility level, leaving us uncertain about the source of variation in the facilities. We measured some socioeconomic factors, including literacy and income, at the district level, potentially masking variation between patients. Geographical location of the study hub was included in the model to understand potential differences in study implementation; however, the central study hub included more facilities than other hubs, which might have introduced bias. We were unable to adjust the birth-volume analysis by the number of staff per facility (to generate a patients-per-capita measure) because we measured staffing once, at the start of the study, rather than on individual days or over time. It is possible that a meaningful reduction in mortality in a setting with a proportion of caesarean-section deliveries of less than 1% is unrealistic and that childbirth at primary-level facilities should be avoided. However, given that a significant percentage of deliveries in Uttar Pradesh occurs at similarly resourced primary and community health facilities, we believe that it is still important to explore how the current system could be improved. More generally, as a post-hoc study, we had an increased risk for false discoveries; to account for this risk in our methods, we applied multiplicity adjustment.

There is no easy answer to improving maternal and newborn health outcomes. First, we need to improve the quality metrics. The relationship between quality of care and mortality is complex and poorly characterised, partly because of the lack of complete and nuanced measurements of quality of care. Second, reducing mortality will require improvements at multiple levels of the health system. Successfully changing birth attendants' behaviour at the facility level might require high-touch, long-term implementation methods, and such behavioural change might prove insufficient without also addressing social determinants of health such as education and poverty. Until we redesign how we measure and seek to improve the quality of care, we will continue to achieve only partial victories in making childbirth safer worldwide.

## Data sharing

The data repository will be available upon publication on Harvard University Dataverse and will include deidentified data and a data dictionary.
